# Strychnine in Fevers

**Published:** 1847-10

**Authors:** A. W. Benton

**Affiliations:** Sterling Ill.


### ARTICLE III.

### STRYCHNINE IN FEVERS.

*Messrs. Editors*:—I understand there is an editorial article in the last Journal, (which I have not seen) on the same, or a similar subject to the heading of this article. The following, however, are the facts which I have been some time in collecting in relation to the use of strychnine to allay certain symptoms in fever.

In the "Bulletin of Medical Science" for May, 1846, is a short article communicated by me on the same subject. Since then I have had more extended experience in its use, and am happy to say it has never disappointed me in its operation.

The particular symptom for which I have given it is coma, as we often see it in our autumnal fevers.

When coma comes on in the course of a fever, without any organic lesion of the nervous centres, I give strychnine with the utmost confidence of its relieving that symptom, if the patient is not already beyond the reach of all medicine. I have never given it in but two cases that terminated fatally; in both the patient came out the coma, and remained so till a few hours before dissolution.

In cases of great languor and lassitude, with a tendency to stupor, it has the happiest effect. It seems to arouse the energies, and give tone and vigor to the whole nervous system, and particularly to that portion which presides over all the voluntary functions.

It seems to prepare the system, (in connection with alteratives,) more effectually, for the successful operation of quinine in permanently breaking up the paroxysms of remittent or intermittent fevers, than any medicine I know of.

To relieve coma, I give it in 1-12 of gr. doses every six hours, and in bad cases every four hours.

The modus operandi of the two articles, strychnine and quinine, in the cure of fevers of the adynamic type seems to me to be as follow: strychnine furnishes the system with the appropriate material for generating the nervous power, and quinine furnishes the blood with the appropriate material for stimulating, or calling that power into action. I have designedly made this article short, supposing that ideas, and not words, are what you most desire for publication. Yours, &c.

A. W. BENTON.

Sterling, Aug. 3, 1837.

